# 1,1,3,3-Tetramethylguanidine-Mediated Zwitterionic Ring-Opening Polymerization of Sarcosine-Derived *N*-Thiocarboxyanhydride toward Well-Defined Polysarcosine

**DOI:** 10.1021/acs.macromol.1c02472

**Published:** 2022-03-30

**Authors:** David Siefker, Brandon A. Chan, Meng Zhang, Ju-Woo Nho, Donghui Zhang

**Affiliations:** Department of Chemistry and Macromolecular Studies Group, Louisiana State University, Baton Rouge, Louisiana 70803, United States

## Abstract

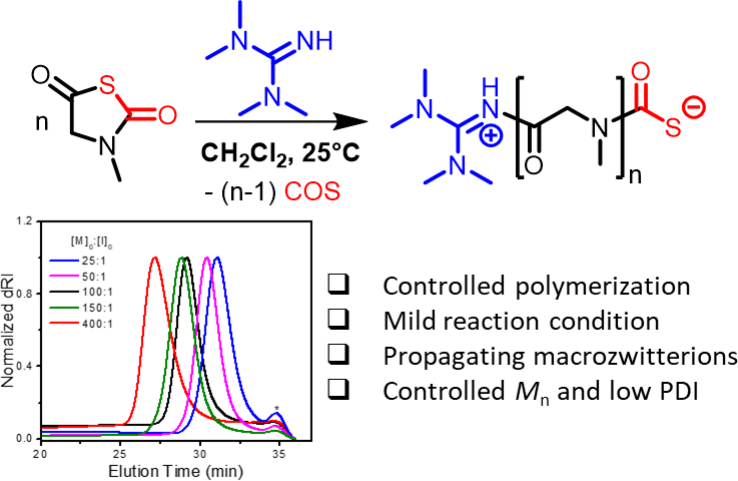

Zwitterionic ring-opening polymerization (ZROP) of sarcosine-derived *N-*thiocarboxyanhydrides (Me-NNTAs) can be induced by using 1,1,3,3-tetramethylguanidine (TMG) initiators in CH_2_Cl_2_ at 25 °C, rapidly producing well-defined polysarcosine polymers with controlled molecular weights (*M*_n_ = 1.9–37 kg/mol) and narrow molecular weight distributions (*Đ* = 1.01–1.12). The reaction exhibits characteristics of a living polymerization, evidenced by pseudo-first-order polymerization kinetics, the linear increase of polymer molecular weight (*M*_n_) with conversion, and the successful chain extension experiments. The polymerization is proposed to proceed via propagating macro-zwitterions bearing a cationic 1,1,3,3-tetramethylguanidinium and an anionic thiocarbamate chain end. The TMG not only initiates the polymerization but also serves to stabilize the thiocarbamate chain end where the monomer addition occurs. Because of the enhanced hydrolytic stability of Me-NNTA, the polymerization can be conducted without the rigorous exclusion of moisture, further enhancing the appeal of the method to access well-defined polysarcosine.

## Introduction

Zwitterionic polymerization proceeds with a zwitterionic propagating species where one chain end is positively charged and the other is negatively charged. Chain elongation occurs either by condensation of the propagating macro-zwitterions in a step-growth fashion or by addition of monomer to the chain end of the macro-zwitterions in a chain growth manner.^1^ Intramolecular or intermolecular end-to-end coupling is a common mode of chain transfer or termination in zwitterionic polymerization. The zwitterionic propagating species can adopt either cyclic or linear architecture, depending on the polymer conformation/chain rigidity, nature of the ionic moieties at the chain ends (thereby the monomer and initiator), and solvent, which modulates the strength of electrostatic interaction among the chain ends.^1−5^ A variety of polar monomers (e.g., cyanoacrylate,^6−9^*N*-substituted maleimide,^10^ methacrylate derivatives,^11^ vinyl ether,^12^ cyclic ester,^5,13−16^ cyclic ether,^3,4,17,18^ cyclic phosphate,^19^ cyclic carbosiloxane,^20^ and cyclic *N*-carboxyanhydride)^21−23^ have been shown to undergo zwitterionic polymerization using either nucleophilic initiators (e.g., tertiary amine, pyridine, phosphine, imidazole, isothiourea, DBU, *N*-heterocyclic carbene, etc.) or electrophilic initiator (e.g., BF_3_, Sc(OTf)_3_, SnBr_4_, etc.). Nucleophilic monomers (e.g., oxazoline, cyclic phosphonite, imino ether, etc.) and electrophilic monomers (e.g., propiolactone, 1,3-propane sultane, acrylic acid, ethylene sulfonamide, acrylamides, etc.) have also been shown to undergo spontaneous zwitterionic copolymerization, producing the respective alternating copolymers.^24−28^

Polysarcosine, a structural analogue of polyalanine, is structurally the simplest polypeptoid, an emerging class of pseudopeptidic polymers featuring *N*-substituted polyglycine backbones.^29,30^ Because of *N*-methyl substitution, polysarcosine is highly water-soluble with a solvated coil chain conformation.^31^ This is distinctly different from analogous polyalanines, which exhibit poor solubility in water and adopt an α-helical conformation.^32^ The strong water solvation and minimal cytotoxicity of polysarcosine make it an attractive surrogate for poly(ethylene glycol) (PEG) for various biomedical and biotechnological applications.^33,34^ Polysarcosine is most commonly obtained by controlled ring-opening polymerization (ROP) of sarcosine-derived *N*-carboxyanhydride (Me-NNCA) monomers using nucleophilic initiators (e.g., primary amine^35^ or *N*-heterocyclic carbene).^21^ Sarcosine-derived *N*-thiocarboxyanhydride (Me-NNTA), the mercapto analogue of the Me-NNCA, has been increasingly scrutinized for polymerization to produce polysarcosine, given the significantly enhanced hydrolytic stability and shelf life of the former relative to the latter.^36^ Primary amine and rare earth metal borohydride initiators have both been shown to successfully initiate the polymerization of Me-NNTA, producing polysarcosine with tailorable molecular weight in acetonitrile (ACN).^37,38^ Polymerization of Me-NNTA using primary amine initiator proceeds by the normal amine mechanism. The slow release of the COS from the thiocarbamate propagating chain end appears to be the rate-limiting step.^39,40^ Free COS in the solution can undergo a side reaction with water,^41^ causing premature termination of chain growth. Controlled polymerization of Me-NNTA in highly polar media (e.g., DMF, NMP, and DMAc) requires the addition of excess weak acid to accelerate the COS release.^40^ All reported syntheses of polysarcosine by polymerization of Me-NNTA require elevated temperature and prolonged reaction time, particularly when high polymer molecular weights are desired.^38,40^

**Scheme 1:**
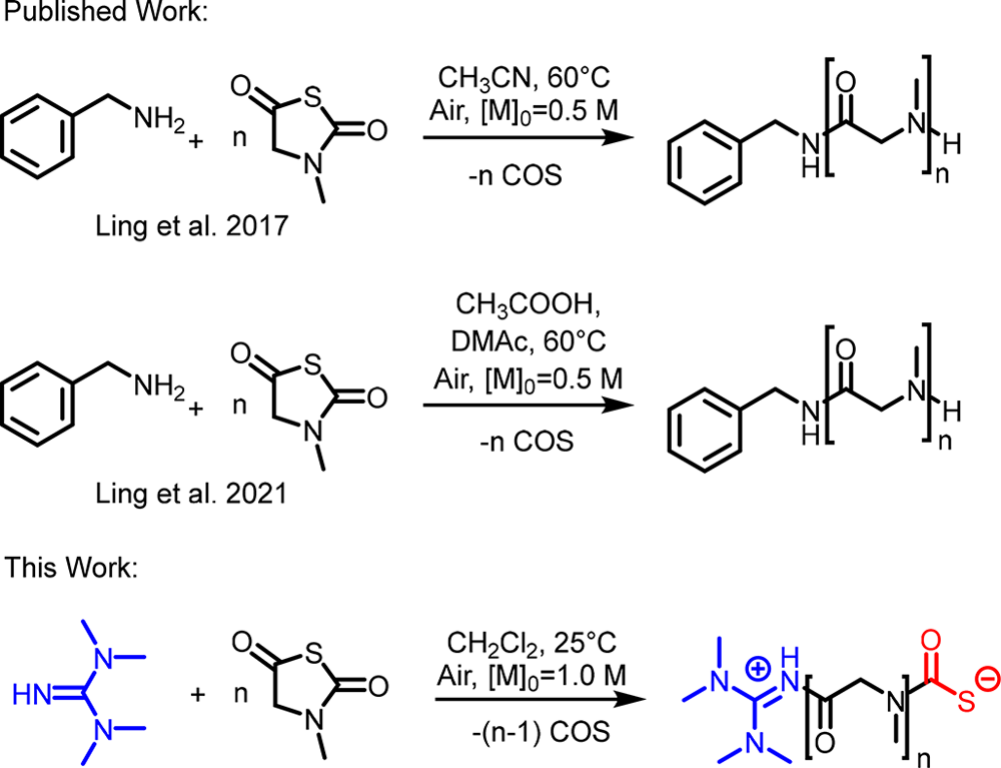


In this contribution, we investigated the ring-opening polymerization of sarcosine-derived *N*-thiocarboxyanhydride (Me-NNTAs) using 1,1,3,3-tetermethylguanidine (TMG) as the initiator. The reaction was shown to exhibit controlled polymerization characteristics and proceed rapidly under mild conditions (25 °C, in CH_2_Cl_2_), producing polysarcosine with tunable molecular weight (*M*_n_ = 1.9–41 kg/mol) and narrow molecular weight distribution (*Đ* = 1.01–1.08) (Scheme 1). A combination of spectroscopic and kinetic analyses revealed that the polymerization occurs via a propagating macro-zwitterion bearing oppositely charged 1,1,3,3-tetramethylguanidinium moiety and thiocarbamate moiety at each chain end. TMG not only initiates the polymerization but also serves to stabilize the thiocarbamate moiety from which monomer addition occurs by the counterion effect.

## Results and Discussion

Me-NNTA monomer was synthesized by following a reported procedure.^37^ The structure and purity of the monomer were confirmed by a combination of ^1^H NMR, ^13^C NMR, and FT-IR spectroscopy (Figures S1–S3). Polymerization of Me-NNTA in the presence of TMG was first screened in different solvents at 25 °C under identical conditions (i.e., [Me-NNTA]_0_ = 1.0 M, [Me-NNTA]_0_:[TMG]_0_ = 160:1). Quantitative conversion was obtained within 5 h for polymerization conducted in CH_2_Cl_2_, in contrast to those in THF or ACN affording partial conversions (Table S1 and Figure S4). As a result, subsequent polymerization studies were all conducted in CH_2_Cl_2_ solvent.

**Figure 1:**
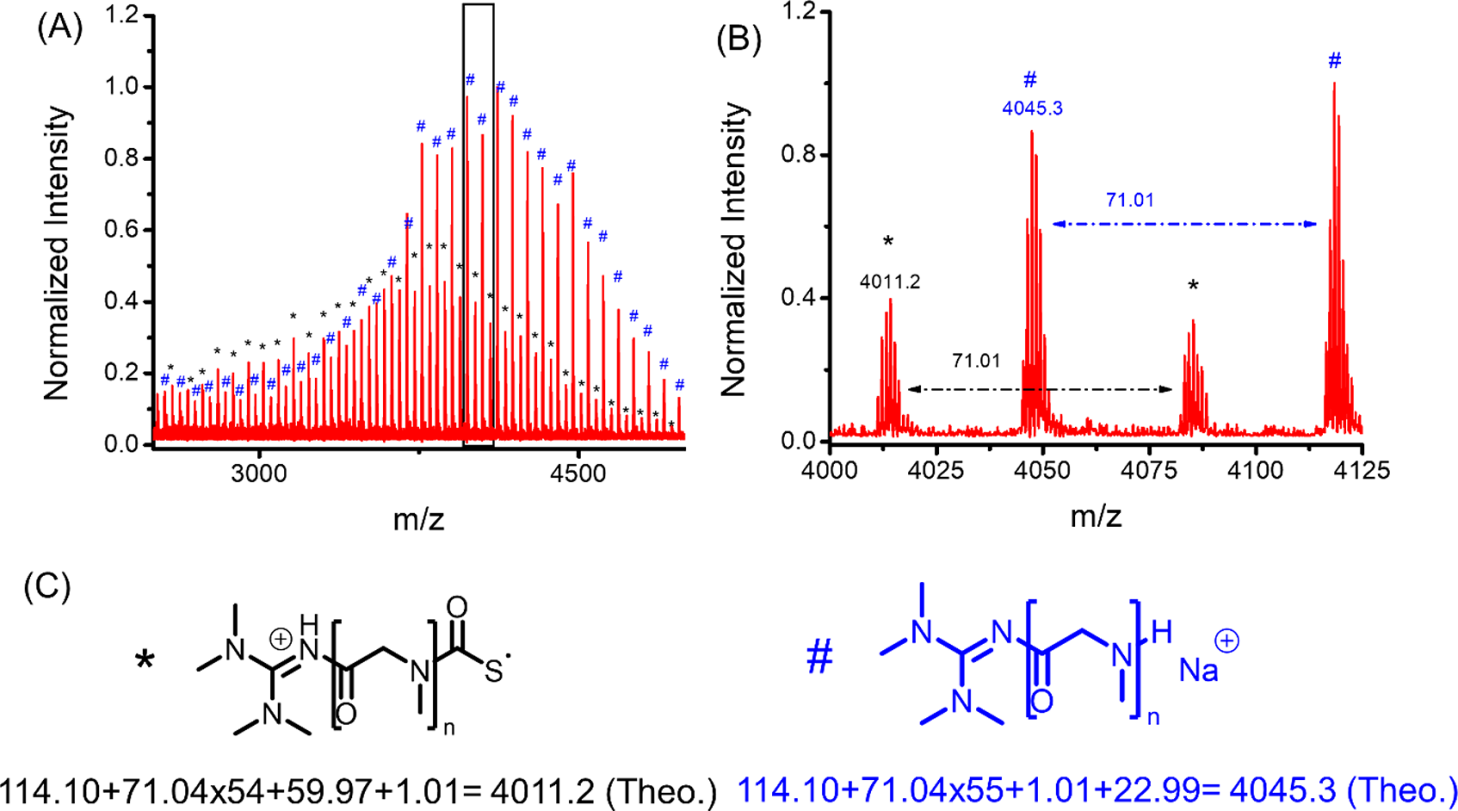
(A) Full and (B) expanded MALDI-TOF MS spectra of a low molecular weight polysarcosine polymers obtained by the TMG-mediated ROP of Me-NNTA ([Me-NNTA]_0_:[TMG]_0_ = 50:1) in CH_2_Cl_2_ at 25 °C and (C) the polymer structures that are consistent with the mass ions in the MS spectrum.

A series of polymerizations of Me-NNTA in the presence of TMG were conducted in CH_2_Cl_2_ at 25 °C with a constant initial monomer concentration ([Me-NNTA]_0_ = 1.0 M) and varying initial monomer-to-initiator molar ratios ([Me-NNTA]_0_:[TMG]_0_ = 25:1–400:1) (Scheme 1). All reactions reached quantitative conversion within 24 h, evidenced by the complete disappearance of FT-IR peak at 1737 cm^–1^ that is characteristic of the Me-NNTA monomer (Figure S5). The polymers were fully characterized by MALDI-TOF MS, ^1^H and ^13^C NMR, and size-exclusion chromatography coupled to a multiangle-light scattering and a differential refractive index detector (SEC-MALS-DRI). ^1^H and ^13^C NMR analyses confirmed the desired polysarcosine backbone structure and the presence of a cationic TMG moiety and an anionic thiocarbamate moiety affixed to the polymer chain (Figures S6–S8). The polymer structure is further corroborated by the 2D HMBC and HSQC NMR analysis (Figures S9 and S10). MALDI-TOF MS analysis of the low molecular weight polymer revealed the presence of molecular ions that are consistent with the polysarcosine polymers bearing a cationic TMG moiety and a thiocarbamate-derived radical moiety at each chain end (Figure 1), consistent with initiation of polymerization by nucleophilic addition of TMG to Me-NNTA. Note that the formation mechanism of the observed polysarcosine species bearing the thiocarbamate-derived radical chain end is not entirely clear (Figure 1C). It is presumed to have formed by laser irradiation during the MALDI-TOF MS experiment. In addition, polysarcosine polymer bearing a neutral TMG moiety and a secondary amino chain end was observed (Figure 1), which is presumed to form in the presence of exogeneous protic acid during the MALDI-TOF MS sample preparation (*vide infra*).

**Figure 2:**
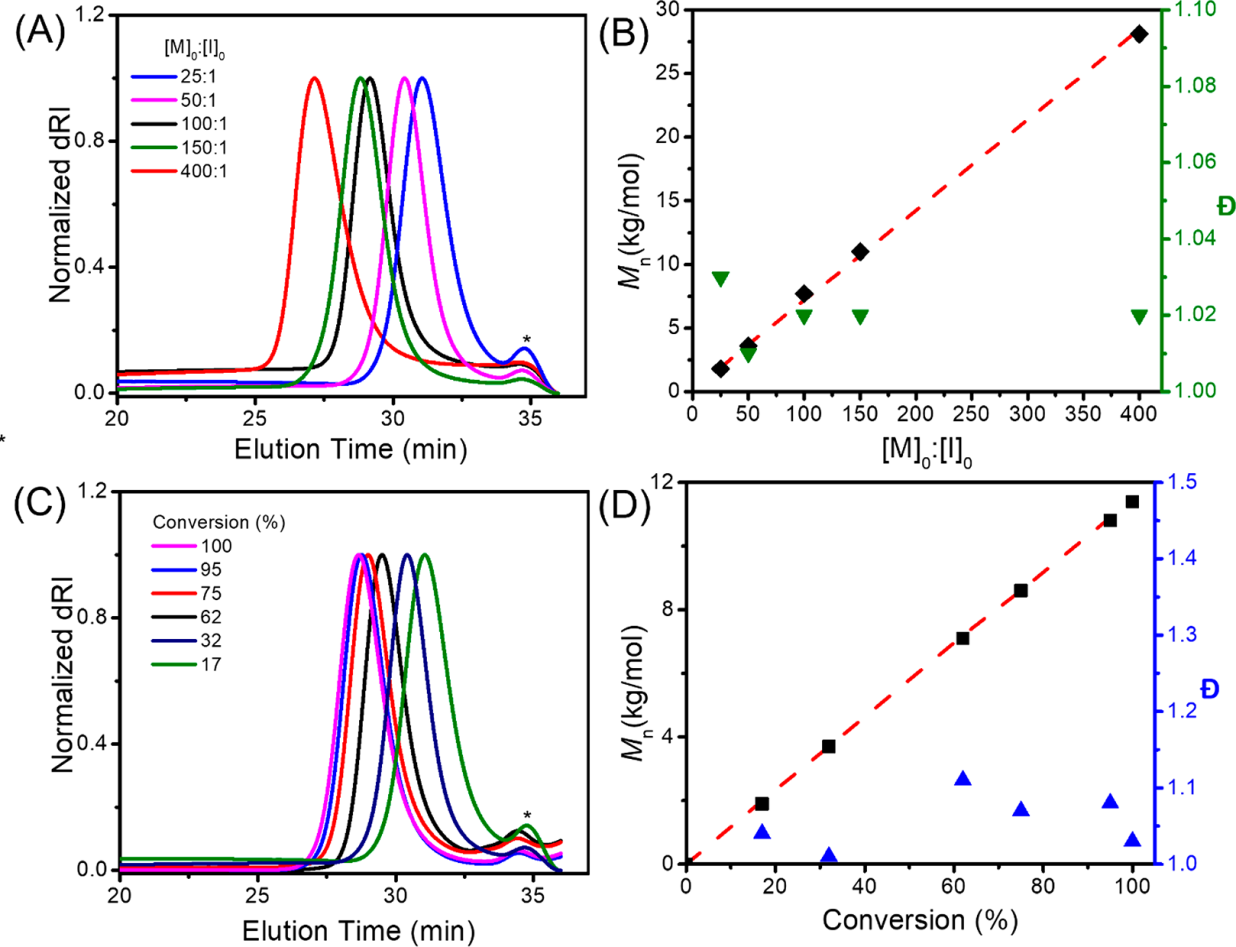
(A) Representative SEC chromatograms of polysarcosine obtained by ROPs of Me-NNTA using TMG initiators with varying initial monomer-to-initiator ratios ([M]_0_:[I]_0_) after reaching quantitative conversion in 1–20 h (conditions: [M]_0_ = 1.0 M, [I]_0_ = 40, 20, 10, 6.7, and 2.5 mM). (B) Plots of *M*_n_ (SEC) (◆), *M*_n_ (Theor.) (---) and *Đ* (▼) versus [M]_0_:[I]_0_ for the ROP of Me-NNTA using the TMG initiator (conditions: [M]_0_ = 1.0 M, [I]_0_ = 40, 20, 10, 6.7, and 2.5 mM). (C) Representative SEC chromatograms of polysarcosine obtained by ROPs of Me-NNTA using TMG initiators at different conversions (conditions: [M]_0_ = 1.0 M, [M]_0_:[I]_0_ = 160:1). (D) Plots of *M*_n_ (■), *M*_n_(theor) (---) and *Đ* (▲) versus conversion for the ROP of Me-NNTA using the TMG initiator ([M]_0_ = 1.0 M, [M]_0_:[I]_0_ = 160:1). All reactions were conducted at 25 °C in CH_2_Cl_2_ unless otherwise noted. The peaks (∗) eluted at ∼35 min in the SEC chromatograms (A and C) are from the solvent.

SEC-MALS-DRI analysis revealed monomodal distribution of the polysarcosine polymers with *M*_n_ value in the 1.9–28.1 kg/mol range that can be controlled by adjusting the initial monomer-to-TMG ratios (Table 1). The polymer molecular weight distribution remains narrow (*Đ* = 1.01–1.03) in the entire molecular weight range (Figure 2A,B). Furthermore, the experimental polymer molecular weights (*M*_n_) determined by the SEC analyses agree well with the theoretical values based on a single-site initiation by TMG (Table 1 and Figure 2B). In addition, the experimental polymer molecular weight (*M*_n_) was also found to increase linearly with conversion for the TMG-mediated polymerization of Me-NNTA in CH_2_Cl_2_ at 25 °C, indicating a constant concentration of propagating species throughout the course of the reaction. The molecular weight distribution remains narrow (*Đ* = 1.003–1.13) throughout the entire polymerization (Figure 2C,D). ^1^H NMR analysis of the polymerization reaction mixture revealed the absence of any free TMG initiator, suggesting quantitative incorporation of TMG into the polymer chain through initiation.

**Table 1:** Ring-Opening Polymerization of Me-NNTA Using the TMG Initiator^a^

entry no.	[I]_0_	[M]_0_:[I]_0_	*M*_n_ (Theor.)^b^ (kg/mol)	*M*_n_(SEC)^c^ (kg/mol)	*Đ*^c^
1	TMG	25:1	1.8	1.9	1.03
2	TMG	50:1	3.6	4.1	1.01
3	TMG	100:1	7.2	7.7	1.02
4	TMG	150:1	10.7	11.0	1.02
5	TMG	400:1	28.4	28.1	1.02

a All polymerizations were conducted with [M]_0_ = 1.0 M in CH_2_Cl_2_ at 25 °C and reached quantitative conversion in 24 h.

b Theoretical *M*_n_ was calculated based on [M]_0_:[I]_0_ ratios and quantitative conversion.

c *M*_n_(SEC) and polydispersity index were determined by SEC-MALS-DRI in HFIP/CF_3_CO_2_K (3 mg/mL) at 40 °C by using d*n*/d*c* = 0.23 mL/g.

**Figure 3:**
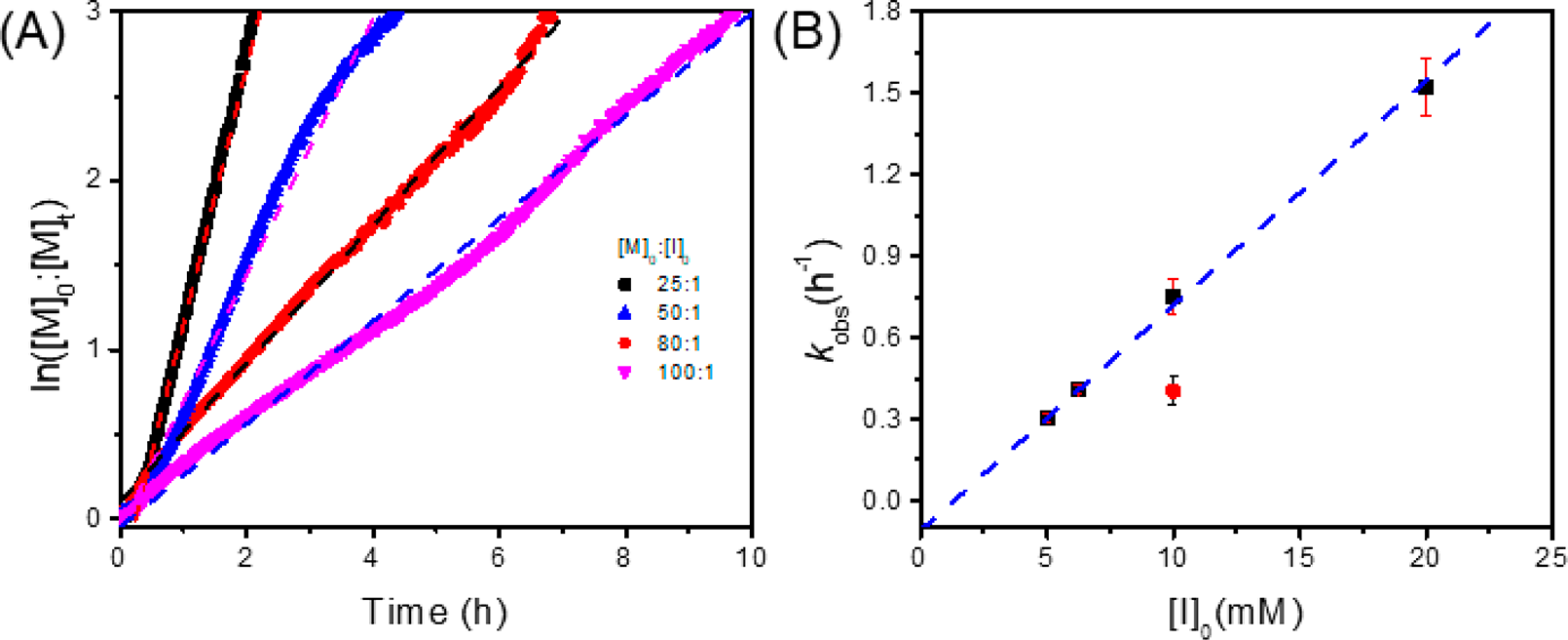
(A) Plots of ln([M]_0_:[M]_*t*_) versus time for ROPs of Me-NNTA using TMG initiators with varying initial monomer-to-initiator ratio ([M]_0_:[I]_0_ = 25:1 (■), 50:1 (▲), 80:1 (●), and 100:1 (▼)) and linear fitting of the data (---) (*k*_obs_ = 1.5 ± 0.1, 0.75 ± 0.07, 0.40 ± 0.01, and 0.30 ± 0.01 h^–1^). (B) Plot of observed polymerization rate constant (*k*_obs_) versus [I]_0_ for ROPs of Me-NNTA using the TMG (■) or ^n^BuNH_2_ initiator (●) (*k*_obs_ = 0.40 ± 0.05 h^–1^, [M]_0_:[I]_0_ = 80:1) and the linear fitting of the data with the TMG initiator (---) (*k*_p_ = 83 ± 3 M^–1^ h^–1^). All reactions were conducted with a constant initial monomer concentration ([M]_0_ = 0.5 M) at 25 °C in CH_2_Cl_2_.

Kinetic studies were conducted for the TMG-mediated polymerization of Me-NNTA in CH_2_Cl_2_ at 25 °C with a constant initial monomer concentration ([M]_0_ = 0.5 M) and varying initial monomer-to-initiator ratio ([M]_0_:[I]_0_ = 25:1–100:1) (Figure 3A). The plots of ln([M]_0_:[M]_*t*_) versus time pass through (0,0) following a linear relationship (Figure 3B), consistent with a first-order dependence of polymerization rate on the monomer concentration with the observed rate constant (*k*_obs_) in the 1.5 ± 0.1 to 0.30 ± 0.01 h^–1^ range for varying initiator loading ([I]_0_ = 5–20 mM). The plots *k*_obs_ versus initial TMG concentrations also afforded a linear relationship, indicating a first-order dependence of the polymerization rate on the initiator concentration with the polymerization rate constant (*k*_p_) of 83 ± 3 M^–1^ h^–1^. In addition, the polymerization of Me-NNTA using the TMG initiator is nearly twice as fast as that conducted by using the *n*-butylamine initiator under otherwise identical conditions ([M]_0_:[I]_0_ = 80:1, Figure 3B), suggesting that these two polymerizations are likely to occur by different mechanisms (*vide infra*). In addition, it should be noted that the rate of polymerization of Me-NNTA using TMG initiators in 25 °C CH_2_Cl_2_ is comparable to that using benzylamine initiators in ACN solvent at elevated temperature (70 °C)^38^ and the benzylamine-initiated polymerization of the more reactive Me-NNCA in 20 °C NMP solvent (Table S2).^35^ The choice of solvent and the nature of reactive zwitterionic propagating species are likely the contributing factors to the fast polymerization observed for the Me-NNTA using TMG initiator in CH_2_Cl_2_.

**Figure 4:**
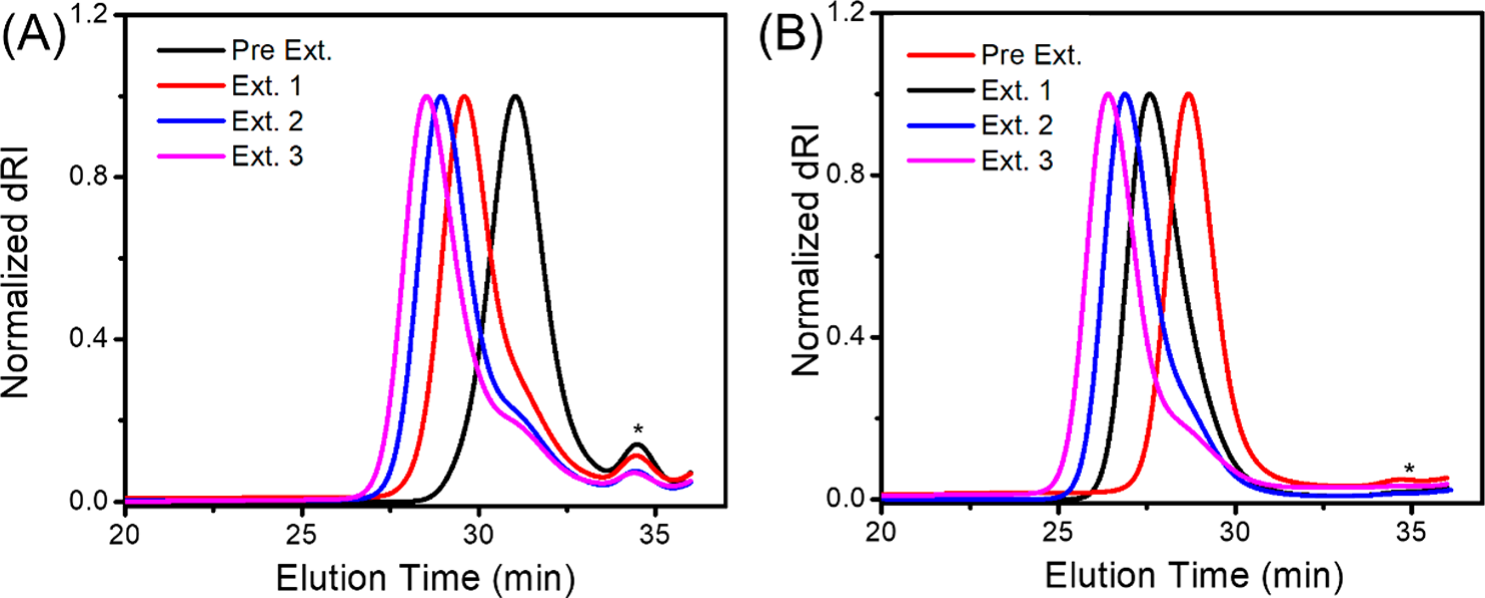
(A) SEC chromatograms of polysarcosine polymers obtained from the chain extension using a low molecular weight polysarcosine macroinitiator (*M*_n_(SEC) = 4.4 kg/mol, *Đ* = 1.08) or (B) a high molecular weight polysarcosine macroinitiator (*M*_n_(SEC) = 10.8 kg/mol, *Đ* = 1.004). The macroinitiator was formed *in situ* by TMG-initiated ROP of Me-NNTA in CH_2_Cl_2_ at 25 °C and used directly in chain extension experiments. All chain extension reactions were allowed to reach quantitative conversion prior to new monomer addition. The peaks (∗) eluted at ∼35 min in the SEC chromatograms are from the solvent.

Chain extension was also conducted by using a high (*M*_n_(SEC) = 10.8 kg/mol, *Đ* = 1.004) or a low molecular weight polysarcosine macroinitiator (*M*_n_(SEC) = 4.4 kg/mol, *Đ* = 1.08) formed *in situ* by the TMG-mediated ROP of Me-NNTA in CH_2_Cl_2_ at 25 °C. Additional three batches of Me-NNTA ([M]_0_:[PNMG macroinitiator]_0_ = 150:1) were sequentially introduced into the polysarcosine macroinitiator solution in CH_2_Cl_2_ to allow for chain extension at 25 °C. Each chain extension was allowed to reach quantitative conversion. SEC analysis revealed a systematic increase of molecular weight of the polysarcosine polymer with low-to-moderate polydispersity (*Đ* = 1.03–1.12) formed from each chain extension (Figure 4). In addition, the *M*_n_ of the polysarcosine polymers resulted from each chain extension reaction agrees reasonably well with the theoretical value assuming quantitative chain extension at the low-to-intermediate molecular weight range (*M*_n_ < 30 kg/mol) (Table 2). There is a more notable deviation of experimental *M*_n_(SEC) from the theoretical value at high molecular weight range (*M*_n_ > 30 kg/mol) (Table 2). It should be noted that a small shoulder at short elution time became visible in the SEC chromatogram after third chain extension reaction. This suggests the presence of chain transfer or termination event whose effect on the polymer molecular weight distribution becomes more pronounced after multiple chain extensions.

**Table 2:** Molecular Weight and Polydispersity of Polysarcosine Polymers Obtained from Chain Extension Experiment Using a Low or High Molecular Weight Polysarcosine Macroinitiator (*M*_n_(SEC) = 4.4 kg/mol, *Đ* = 1.08; *M*_n_(SEC) = 10.8 kg/mol, *Đ* = 1.004), Respectively

extension no.	*M*_n_ (Theor.)^c^ (kg/mol)	*M*_n_(SEC)^d^ (kg/mol)	*Đ*^d^
1^a^	5.8	6.4	1.03
2^a^	8.6	8.2	1.08
3^a^	10.8	9.5	1.12
1^b^	21.4	21.2	1.03
2^b^	32.1	30.6	1.05
3^b^	42.8	36.9	1.06

a Chain extension was conducted by using a low molecular weight polysarcosine macroinitiator (*M*_n_(SEC) = 4.4 kg/mol, *Đ* = 1.08) (conditions: [M]_0_ = 1.0 M, [M]_0_:[polysarcosine macroinitiator]_0_ = 30:1).

b Chain extension was conducted by using a high molecular weight polysarcosine macroinitiator (*M*_n_(SEC) = 10.8 kg/mol, *Đ* = 1.004) (conditions: [M]_0_ = 1.0 M, [M]_0_:[polysarcosine macroinitiator]_0_ = 150:1).

c Theoretical *M*_n_ was calculated based on the cumulative [M]_0_:[polysarcosine macroinitiator]_0_ ratio and *M*_n_ of the polysarcosine macroinitiators.

d *M*_n_(SEC)s and polydispersity indexes were determined by SEC-MALS-DRI in HFIP/CF_3_CO_2_K (3 mg/mL) at 40 °C using d*n*/d*c* = 0.23 mL/g.

**Scheme 2:**
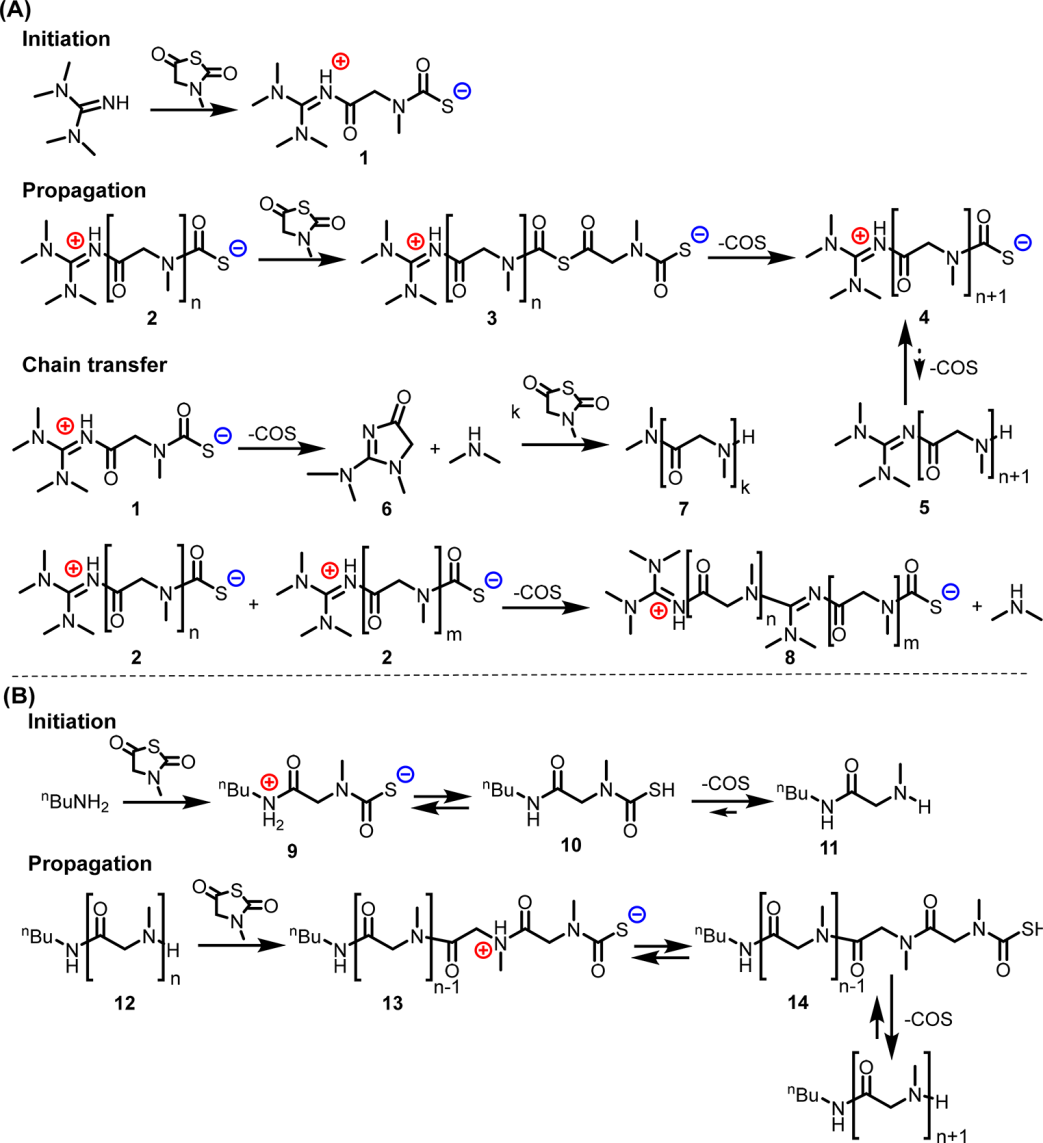
Proposed Reaction Mechanisms for the ROP of Me-NNTA Using (A) TMG versus (B) Primary Amine Initiators

To further investigate the potential chain transfer or termination event, stoichiometric reactions between the Me-NNTA and TMG in 1:1 or 1:5 molar ratio were conducted at 25 °C in CD_2_Cl_2_. A combination of ESI-MS, FTIR, and ^13^C NMR analyses of the reaction mixture (Figures S11–S14) revealed a chain transfer mechanism by either intramolecular transamination of the zwitterionic initiating species **1** or intermolecular transamination involving the zwitterionic propagating species **2**, evidenced by the formation of *N*,*N*-dimethylcreatine **6**, polysarcosine species **7** with an *N*,*N*-dimethylamide chain end, and the zwitterionic polysarcosine species **8** (Scheme 2, Figures S11 and S12).^42,43^ There is no evidence for the formation of macrocyclic polysarcosine species beyond the five-membered cyclic species **6** (*N*,*N*-dimethylcreatine) from the 1:1 Me-NNTA and TMG reaction. Considering that the polysarcosine polymers with a broad range of molecular weight or degree of polymerization (DP_n_ = 25–400) and narrow molecular weight distribution can be obtained by controlling the feed ratio of Me-NNTA relative to TMG initiator in the single batch reaction (Table 1), the rate of chain transfer must be slow relative to the chain propagation. To further assess the extent of intermolecular coupling of propagating macro-zwitterions **2** via transamination (Scheme 2), polysarcosine polymers synthesized by TMG-mediated ROP of Me-NNTA were allowed to stand in CH_2_Cl_2_ at 25 or 50 °C for an additional 72 h after full conversion was reached. SEC analysis revealed an increase of polymer molecular weight and slight broadening of molecular weight distribution for the polymer obtained 1 h after reaching full conversion in 6 h (*M*_n_ = 8.5 kg/mol, *Đ* = 1.02) relative to those allowed to stand for prolonged reaction time (*M*_n_ = 10.2–10.5 kg/mol, *Đ* = 1.07–1.08) (Figure S15 and Table S3), indicating that intermolecular coupling by transamination can occur albeit not to a significant extent relative to chain propagation.

On the basis of the above results, we propose that the TMG-mediated polymerization of Me-NNTA is initiated by ring-opening addition of Me-NNTA with TMG to form a zwitterionic initiating species bearing an acyl guanidinium and a thiocarbamate chain end **1** (Scheme 2A). The propagation entails addition of Me-NNTA onto the thiocarbamate chain end of the propagating macro-zwitterions **2** to form an acyclic thioanhydride intermediate **3** followed by an intramolecular skeletal rearrangement to form the secondary amide linkages accompanied by COS elimination (Scheme 2).^44,45^ This mechanism is akin to that proposed for the ring-opening polymerization of amino acid derived NCAs using aprotic initiators (e.g., pyridine or NaOMe) where a carbamate propagating species was invoked.^23,46^ Our proposed mechanism for the TMG-mediated polymerization of Me-NNTA is based on the following considerations. First, the rate of polymerization of Me-NNTA using TMG initiators is faster than that using ^n^BuNH_2_ initiators by nearly twofold (Figure 3B), suggesting the nature of propagating species is different in these two reactions (**2**, Scheme 2A vs **12**, Scheme 2B). Second, a variety of organic salts composed of amidinium and guanidinium carbamate (^+^RNHCO_2_^–^) or dithiocarbamate (^+^RNHCS_2_^–^) were found to be stable at room temperature, suggesting favorable interactions among these specific organic ion pairs.^47,48^ The positively charged acyl guanidinium chain end in the zwitterionic initiating/propagating species **1**/**2** is conceivably much more stable than the protonated primary/secondary amide moieties of the initiating/propagating intermediates **9**/**13** formed in the primary amine-initiated polymerization of Me-NNTA due to significant resonance stabilization in the former than the latter. As a result, the propagating macro-zwitterions **2** do not readily convert to the neutral species **5**, as the elimination of COS requires proton transfer. In addition, the proton-transfer-assisted dethiocarboxylation (COS release) of thiocarbamate species is known to be more retarded relative to the analogous decarboxylation of carbamate moieties,^49^ which contributes to the persistence of the propagating macro-zwitterion **2** in the TMG-mediated ROP of Me-NNTA (Scheme 2).

## Conclusions

TMG-mediated polymerization of Me-NNTA has been shown to occur rapidly under mild conditions. The reaction exhibits controlled polymerization characteristics, producing well-defined polysarcosine polymers with predictable molecular weights in the 1.9–37 kg/mol range and narrow molecular weight distributions (*Đ* < 1.12). The polymerization was shown to proceed by a zwitterionic propagating species, which differs from that of primary amine-initiated polymerization of Me-NNTA. The living nature of the propagating species allows for multiple chain extensions with good control of resulting polymer molecular weight, making it useful for synthesizing block copolymers. Considering the enhanced hydrolytic stability of NTAs and commercial availability of TMG, the synthetic method reported here represents an attractive route toward well-defined polysarcosine polymers.

## Supporting Information Available

Experimental procedures, spectroscopic characterization (e.g., ^1^H, ^13^C, HSQC and HMBC NMR, FT-IR, ESI MS) of Me-NNTA monomer, polysarcosine polymer, reaction mixture of Me-NNTA and TMG at 1:1 or 5:1 molar ratio, and additional SEC chromatograms of the polysarcosine polymers (PDF)
