# Supplementary material for: 1,1,3,3-Tetramethylguanidine-Mediated Zwitterionic Ring-Opening Polymerization of Sarcosine-Derived N-Thiocarboxyanhydride toward Well-Defined Polysarcosine
Source: Macromolecules. 2022 Mar 30;55(7):2509–16. doi: 10.1021/acs.macromol.1c02472 (PMC9011146; doi:10.1021/acs.macromol.1c02472)
Supplement: Supplementary file 1 — ma1c02472_si_001.pdf [file ma1c02472_si_001.pdf]

Supporting Information

1,1,3,3-Tetramethylguanidine-Mediated Zwitterionic Ring-Opening Polymerization of Sarcosine-Derived *N*-Thiocarboxyanhydride towards Well-Defined Polysarcosine

David Siefker[a#], Brandon A. Chan[a$], Meng Zhang[a], Ju-Woo Nho[a&], and Donghui Zhang[a*]

[a] *Department of Chemistry and Macromolecular Studies Group, Louisiana State University, Baton Rouge, LA 70803*

**Corresponds to*: Donghui Zhang (dhzhang@lsu.edu)

Materials

All chemicals were purchased from VWR and used as received unless specified. Methylene Chloride-D_2 (99.5%) was purchased from Cambridge Isotope. Potassium ethyl xanthate (98%) was purchased from Bean Town Chemicals. Phosphorus trichloride (98%) was purchased from Alpha Aesar. All reagents were used as received unless otherwise specified. All solvents (*e.g.*, CH_2Cl_2, THF, ACN) were regular ACS grade solvents and used directly in the reactions without any special drying or purification step unless specified.

Instrumentation

SEC analysis was performed using a Tosoh Bioscience EcoSEC system (HLC 8230 GPC model, Tosoh Bioscience degasser, isocratic pump, autosampler, and column heater) equipped with two TSKgel Alpha-M 13 µm, 7.8 mm I.D. × 30 cm columns, a Tosoh Bioscience dual flow RI detector with a 630–670 nm LED light source, and a Tosoh Bioscience LenS3 multiangle light scattering (MALS) detector (30 mW diode laser at λ = 505 nm). HFIP with 3 mg/mL CF_3CO_2K was used as the eluent at a flow rate of 0.45 mL/min. The pump housing, column oven, and RI detector temperatures were set at 40 °C. All data analysis was performed using SECview software. Polymer molecular weight and molecular weight distribution were obtained by analyzing the RALS-DRI data based on the LS and RI instrument constants that were calibrated with a PMMA standard (M_w(LS) = 32350 g/mol, PDI = 1.03) in HFIP/CF_3CO_2K (3 mg/mL) with known concentration. The refractive index increment (dn/dc) of the polymer was determined to be 0.230 mL/g in HFIP/CF_3CO_2K (3 mg/mL) at 40 °C. Prior to injection into the SEC column, all sample solutions were filtered through 0.45 µm PTFE filters. MALDI-TOF MS experiments

were conducted on a Bruker UltrafleXtreme tandem time-of-flight (TOF) mass spectrometer. The instrument was calibrated with PolyAlanine with a molecular weight range of 600-5000 Da at 30% energy prior to experiment. A saturated methanol solution of α-cyano-4-hydroxycinnamic acid was used as a matrix. Samples were prepared by mixing with a MeCN (0.1% formic acid) solution of polymers (5 mg/mL) with a matrix at a 1:1 volume ratio, followed by the deposition of a few drops (2 μL) of the solution mixture onto a 384-well ground-steel sample plate and drying in air. Experiments were done in positive reflector mode. FTIR spectra for kinetics were collected on a MettlerToledo ReactIR model 45m equipped with a liquid-nitrogen cooled MCT detector. This was connected to an AgX fiber optic conduit to a Mettler-Toledo/Parr high pressure IR cell that used a SiComp (silicon ATR) Sentinel probe. The headpiece of the IR cell was modified with Swaglok quick-connects equipped with solvent-resistant Markez O-rings to facilitate assembly and cleaning. Mettler-Toledo iC IR v7.0 software was used for data collection. FTIR spectra to track conversion were recorded on a Bruker ALPHA II FTIR spectrometer equipped with Platinum ATR. Data were processed using OPUS v7.2 software. All ^{1}H and ^{13}C NMR spectra were recorded using a Bruker AV 500 spectrometer at 298 K. Chemical shifts (δ) are repored in parts per million (ppm) relative to the protio impurities or ^{13}C isotopes in CD_2Cl_2 (or $CDCl_3$) solvent that appear at 5.32 (or 7.26) ppm in ^{1}H NMR spectra and 54.00 (or 77.00) ppm in ^{13}C NMR spectra, respectively.

Methods and Procedures

Scheme S1.

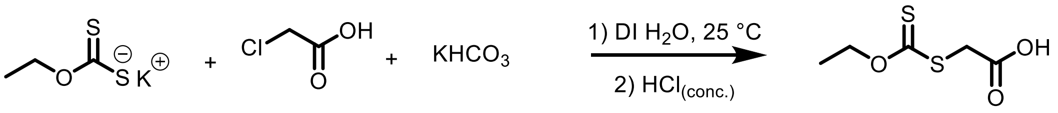

Synthesis of S-Ethoxythiocarbonyl Mercaptoacetic Acid (XAA). The synthesis of XAA is modified from a published procedure.[S1] Potassium ethyl xanthogenoate (18.9 g, 117 mmol) and chloroacetic acid (11.6 g, 117 mmol) were placed in a round bottom flask equipped with a stir bar and dissolved using DI H_2O to afford a 1.0 M solution. The reaction was then cooled to 0°C, and $KHCO_3$ (14.0 g, 140 mmol) was added and dissolved in the solution. The reaction mixture was stirred at 25 °C for 24 h, then acidified to pH= 2 and washed three times with excess CH_2Cl_2 (2.5:1, v/v for each wash). The combined CH_2Cl_2 layer was washed once with DI H_2O, dried over anhydrous $MgSO_4$, filtered, and concentrated under vacuum to give an off-white solid. The solid was vigorously stirred with hexane and then filtered to afford a white crystal product (19.8 g, 94% yield). ^{1}H NMR (500 MHz, $CDCl_3$) δ (ppm): 1.43 (t, 3H, CH_3), 3.97 (s, 2H, CH_2), 4.65 (q, 2H, CH_2)

Scheme S2.

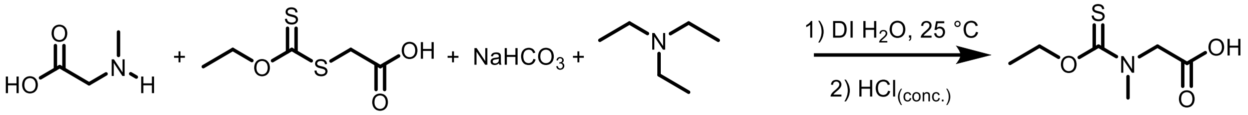

Synthesis of *N*-Ethoxythiocarbonyl Sarcosine (SarXAA). The synthesis of SarXAA is modified from a published procedure.[S2] XAA (13.5 g, 74.3 mmol) and sarcosine (6.62 g, 74.3 mmol) were placed in a round bottom flask equipped with a stir bar and dissolved using DI H_2O to give a 1.0 M solution, to which $NaHCO_3$ (17.1 g, 171 mmol) was subsequently added. In a separate flask, triethylamine (10.3 mL, 74.3 mmol) was dissolved in DI H_2O to give a 1.0 M solution, then added to the above mixture in the round bottom flask and left to react at 25 °C for 24 h. The reaction mixture was then acidified to pH= 2 and extracted three times with excess CH_2Cl_2 (2.5:1, v/v for each extraction). The combined organic layer was washed twice with DI H_2O, 10% citric acid(aq.), and once with DI H_2O. The organic layer was then dried over anhydrous $MgSO_4$, filtered, and concentrated under vacuum to give an off-white solid. The solid was vigorously stirred with hexane and filtered to afford a white crystal product (11.9 g, 90% yield). ^{1}HNMR (500 MHz, $CDCl_3$) δ (ppm): 1.44 (t, 3H, CH_3), 3.19 (s, 3H, CH_3) 3.99 (s, 2H, CH_2), 4.67 (q, 2H, CH_2)

Scheme S3.

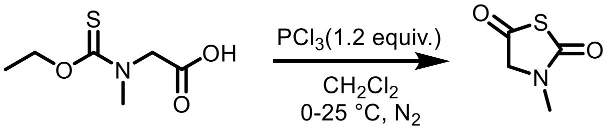

Synthesis of *N*-Methyl-Thiocarboxyanhdrosulfide (Me-NNTA). The synthesis of Me-NNTA follows an adapted, published procedure.[S2] SarXAA (4.78 g, 26.9 mmol) was placed in a dry round bottom flask equipped with a stir bar and dissolved using CH_2Cl_2 to give a 0.5 M solution. The solution was then purged with N_2 at 0 °C for 20 min. PCl_3 (2.8 mL, 32 mmol) was then added to the reaction at 0°C, slowly warmed up to 25 °C, and left to react for 16 h. The reaction was terminated by adding $NaHCO_{3\,(aq.)}$ to pH= 9. Additional CH_2Cl_2 was then added to the reaction mixture. The organic layer was separated, washed with DI H_2O, dried over anhydrous $MgSO_4$, filtered, and concentrated to afford a yellow oil. The oil was then distilled in a vacuum over a short-path distillation apparatus (bath temp. up to 90 °C), producing a clear oil (2.6 g, 74% yield). ^{1}H NMR (500 MHz, CD_2Cl_2) δ (ppm): 3.04 (s, 3H, CH_3), 4.19 (s, 2H, CH_2). ^{1}H{^{13}C}(125 MHz, CD_2Cl_2) δ (ppm): 31.08 (-*C*H_3), 62.21 (-*C*H_2-), 165.02 (-N*C*OS-), 194.57 (-CH_2*C*OS-)

Ring-Opening Polymerization of Me-NNTA using TMG initiators. A representative polymerization procedure is given as follows. Me-NNTA (1.2 g, 9.15 mmol) was dissolved in CH_2Cl_2 (8.9 mL) in a round bottom flask. A measured volume of a stock solution of 1,1,3,3-tetramethylguanidine in CH_2Cl_2 (241 µL, 183 µmol, 75.9 mM) was sequentially added to the above monomer solution at 25 °C then sealed with a rubber septum. The polymerization was

stirred at 25 °C for 2 h. An aliquot of the reaction mixture was taken for conversion analysis. The final polymer product was precipitated by adding excess hexane into the polymerization solution, separated by filtration, and dried under vacuum to afford a white solid (~611 mg, 94% yield).

Chain Extension Experiments. A measured volume of CH_2Cl_2 (227 µL) and a stock solution of 1,1,3,3-tetramethylguanidine in CH_2Cl_2 (23.2 µL, 3.33 µmol, 135 mM) were sequentially added to a stock solution of Me-NNTA (250 µL, 500 µmol, 2.0 M) in CH_2Cl_2 at room temperature in air. The polymerization was stirred at 25 °C for 5 h. An aliquot of the reaction mixture (25 µL) was taken for conversion and molecular weight analysis by FT-IR and SEC methods, respectively. A second batch of Me-NNTA (225 µL, 450 µmol, 2.0 M) was added to the remaining polymerization mixture, which was left to react at 25 °C for an additional 5 h to reach quantitative conversion. The aliquot and addition of 3rd and 4th batch follow same procedure as above. The volatiles were then removed under vacuum to yield a white solid, which was further characterized for conversion and molecular weight by SEC methods.

Kinetic Study of Ring-Opening Polymerization of Me-NNTAs. A representative procedure using TMG initiators is given as followed. A stock solution of Me-NNTA (5 mL, 2.61 mmol, 0.52 M) in CH_2Cl_2 was added to the cell at 25 °C. After initial scans of Me-NNTA in solution, TMG (130 µl, 0.104 mmol, 0.80 M) was added the monomer solution, after which the progression of polymerization was monitored *in situ* by using a Mettler Toledo ReactIR instrument. 256 scans were collected with 1 min interval. Each kinetic experiment was repeated three times to yield the mean observed rate constant (k_{obs}) and the standard deviation.

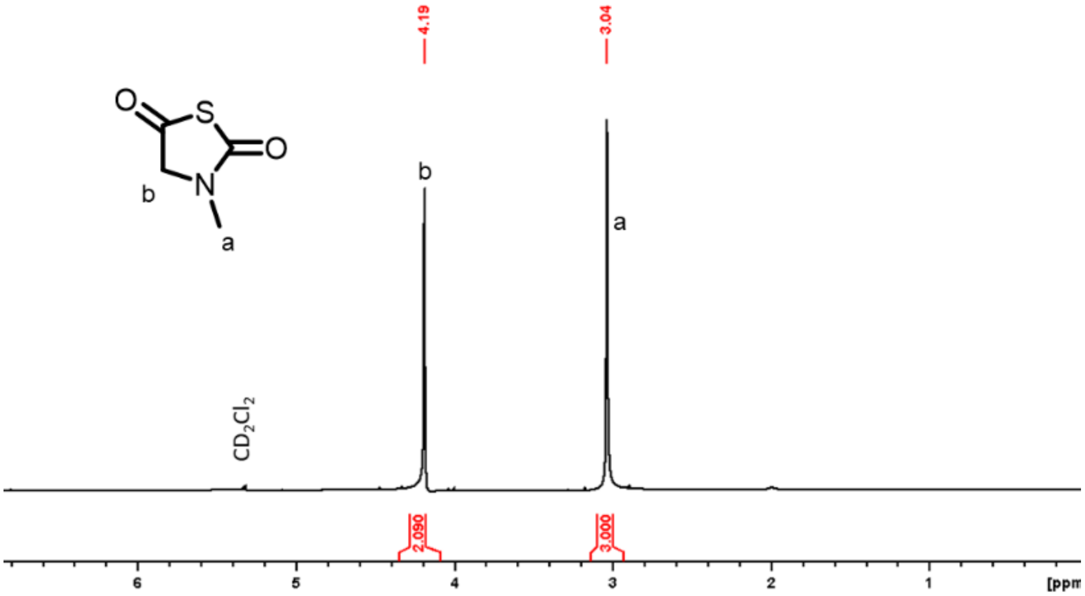

Figure S1. ^{1}H NMR spectrum of Me-NNTA monomers in CD_2Cl_2.

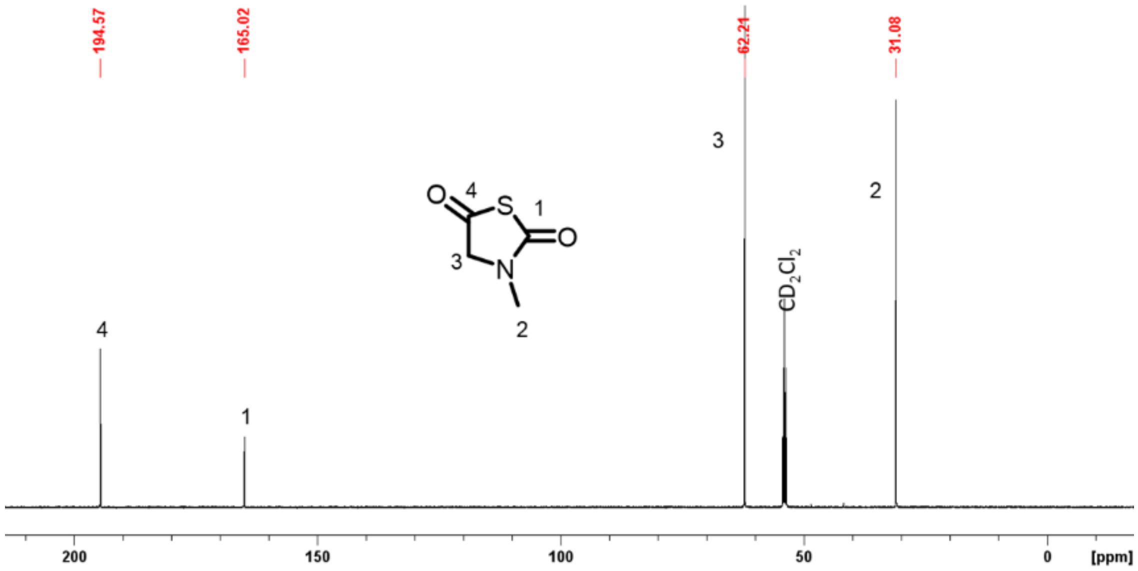

Figure S2. $^{13}C\{^1H\}$ NMR spectrum of Me-NNTA monomer in CD_2Cl_2.

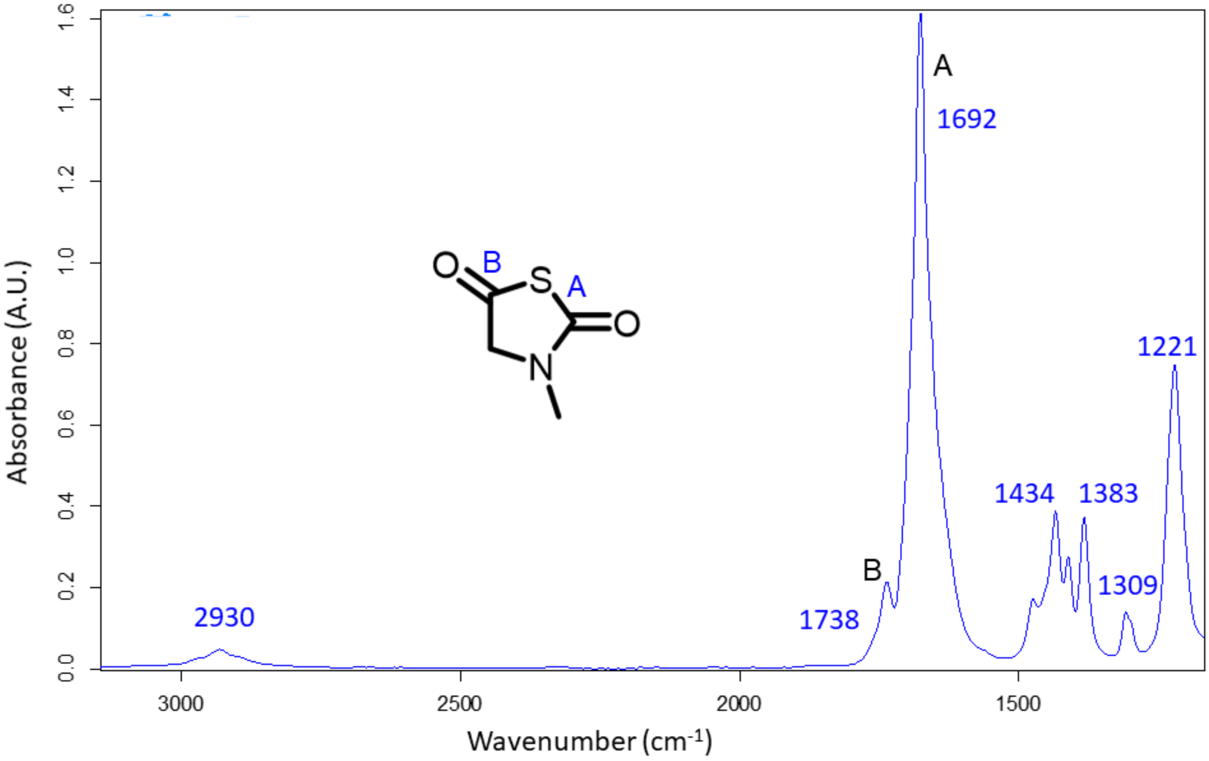

Figure S3. FTIR spectrum of Me-NNTA monomer in CD_2Cl_2.

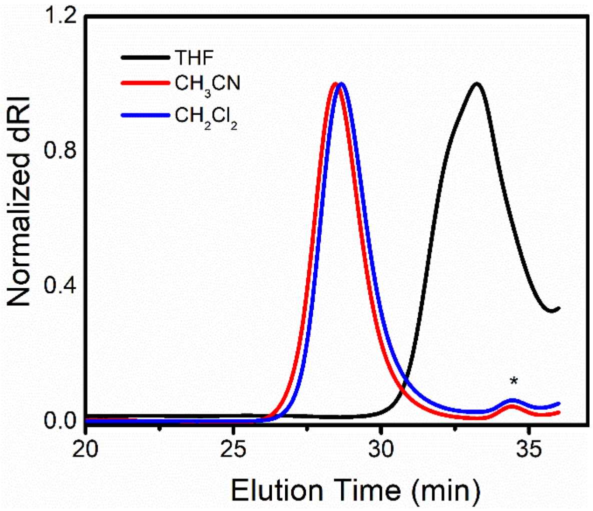

Figure S4. Representative SEC-DRI chromatograms of polysarcosine polymers obtained by TMG-mediated ROPs of Me-NNTA in different solvents (Conditions: $[M]_0$ = 1.0 M, $[I]_0$= 6.25 mM, $[M]_0$:$[TMG]_0$ = 160:1, 25 °C in THF, CH_3CN and CH_2Cl_2, respectively). The SEC chromatograms' peaks (*) eluted at ~35 min are from the solvent.

Table S1. Summary of molecular weight and polydispersity of polysarcosine obtained by TMG-mediated polymerization of Me-NNTA at 25 °C in varying solvents [a]

**Solvent type**	$\boldsymbol{M_n}$ **(Theo.)** [b] **(kg/mol)**	$\boldsymbol{M_n}$ **(SEC)** [c] **(kg/mol)**	**Đ** [c]	**Conversion** [d] **(%)**
CH_2Cl_2	11.5	11.4	1.05	100
CH_3CN	10.9	13.8	1.07	95
THF	6.5	10.5	1.02	57

[a.] All polymerizations were allowed to proceed at 25 °C for 5 h ($[M]_0$=1.0 M, $[I]_0$= 6.25 mM, $[M]_0$:$[TMG]_0$ = 160:1) prior to SEC analysis (**Figure S4**). [b.] Theoretical molecular weights were calculated from $[M]_0$:$[TMG]_0$ ratio and conversion; [c.] Experimental molecular weights and polydispersity index were determined by the SEC-MALS-DRI method using dn/dc = 0.230 mL/g in HFIP/CF_3CO_2K (3 mg/mL) at 40 °C. [d.] Conversion was determined by FT-IR spectroscopy.

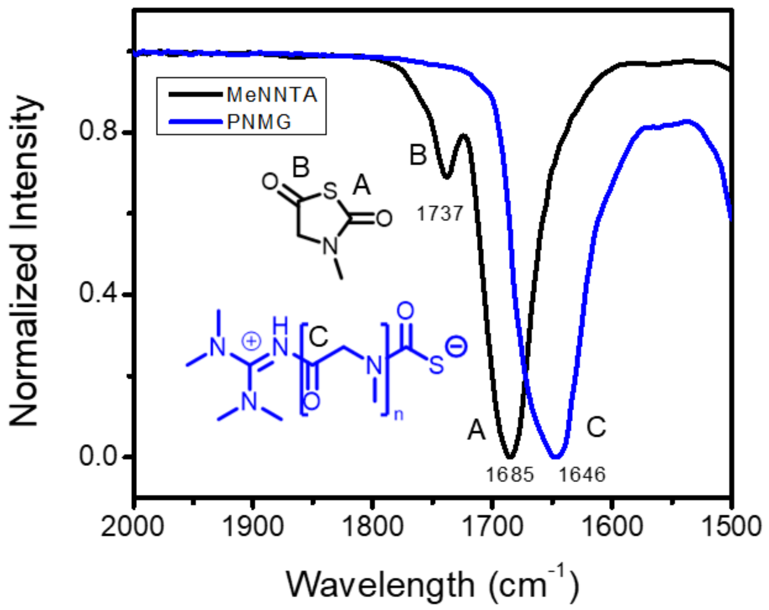

Figure S5. FTIR spectrum of Me-NNTA and polysarcosine polymers (Conditions: $[M]_0$ = 1.0 M, $[I]_0$= 6.25 mM, $[M]_0$:$[TMG]_0$ = 160:1, 25 °C, in CH_2Cl_2).

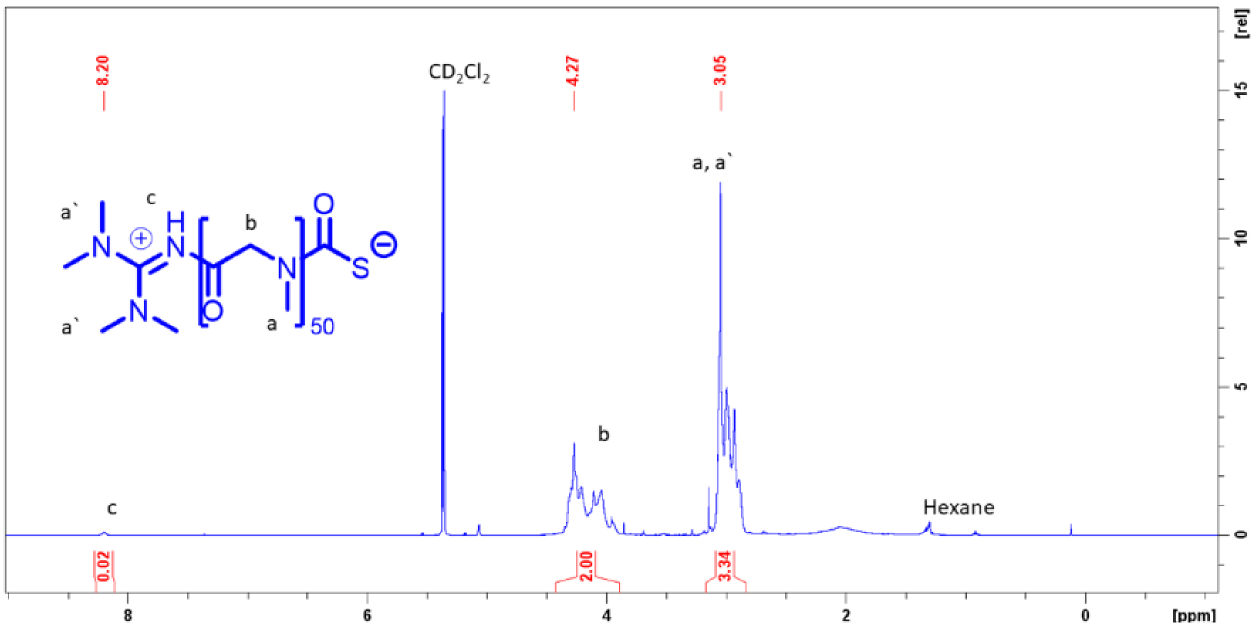

Figure S6. ^{1}H NMR spectrum of polysarcosine polymer in CD_2Cl_2 obtained by TMG-initiated ROP of Me-NNTA and precipitation in hexanes ($[M]_0$ = 1.0 M, $[TMG]_0$ = 20 mM, $[M]_0$:$[TMG]_0$ = 50:1, 25 °C, in CH_2Cl_2).

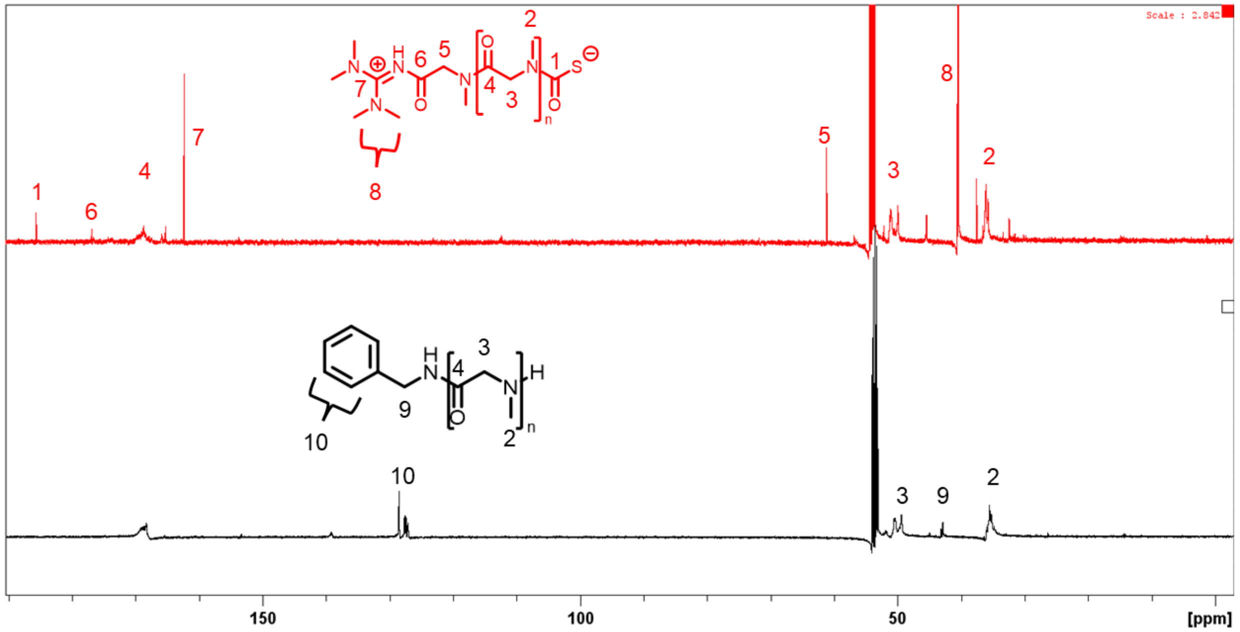

Figure S7. $^{13}C\{^1H\}$ NMR spectrum of 60 mg/mL polysarcosine polymer in CD_2Cl_2 obtained by either TMG or $BnNH_2$-initiated ROPs of Me-NNTA and precipitation in hexanes. ($[M]_0$ = 1.0 M, $[I]_0$ = 100 mM, $[M]_0$:$[I]_0$ = 10:1, 25 °C in CH_2Cl_2).

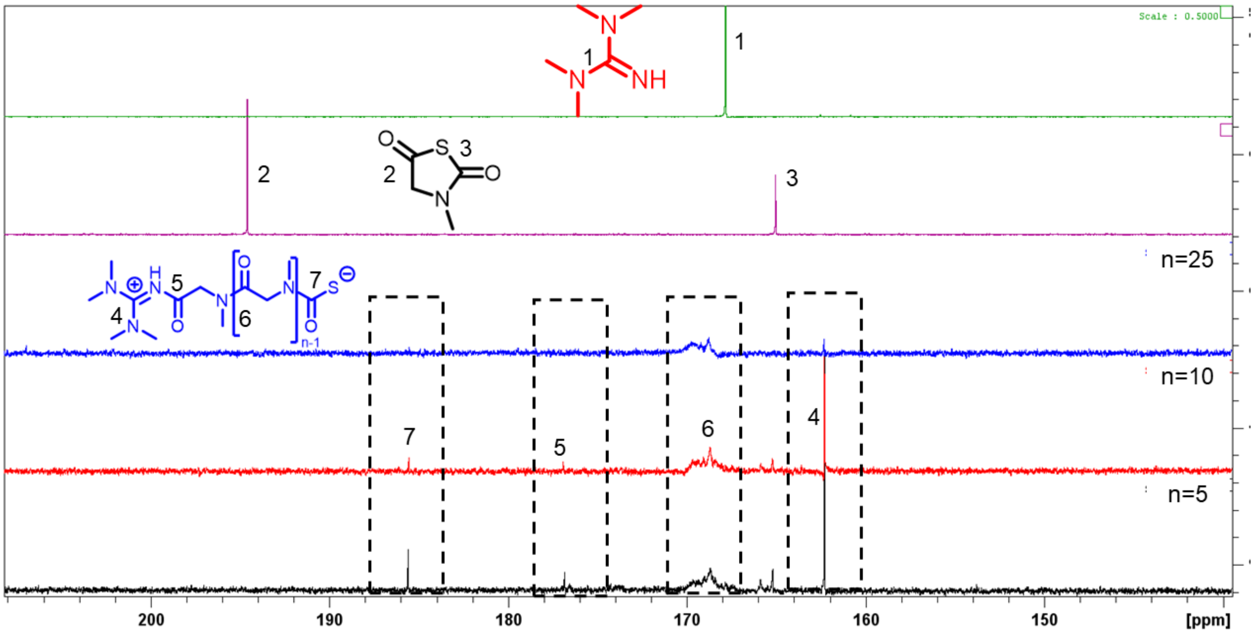

Figure S8. $^{13}C\{^1H\}$ NMR spectra of TMG, Me-NNTA, polysarcosine oligomers obtained by TMG-mediated polymerization of Me-NNTA with varying initial monomer-to-initiator ratio (Condition: $[M]_0$=1.0 M, $[M]_0$:$[TMG]_0$=25:1, 10:1 and 5:1, respectively, 25 °C in CD_2Cl_2). All spectra were collected in CD_2Cl_2 solvent.

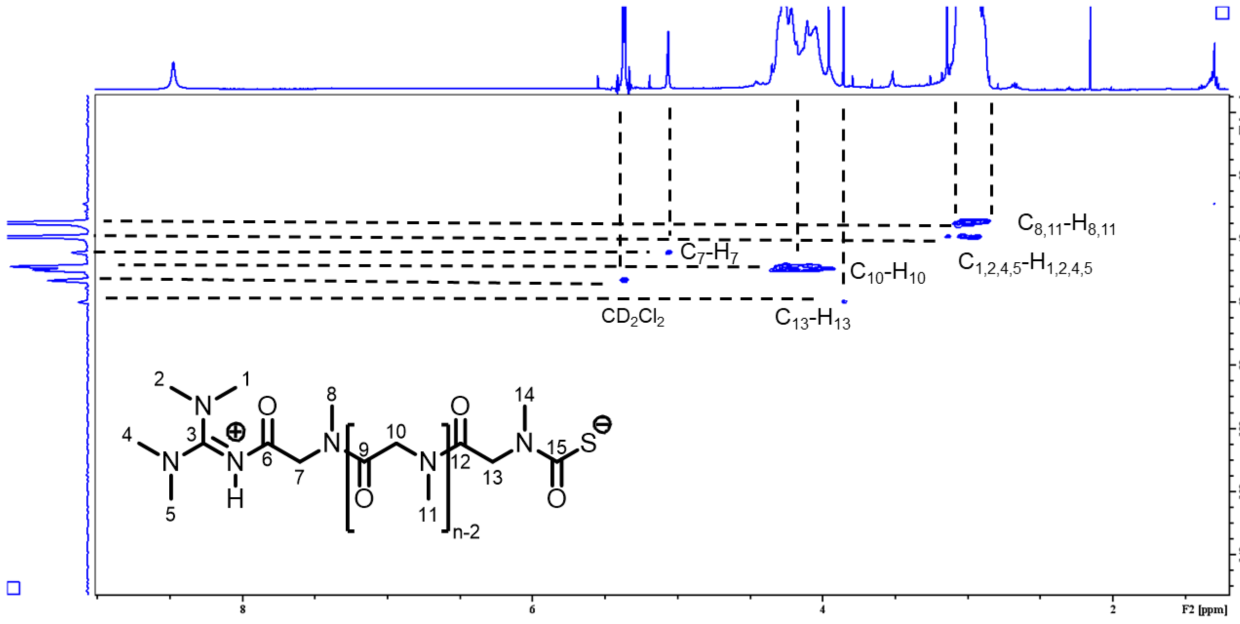

Figure S9. ^{1}H-^{13}C HSQC NMR spectrum of polysarcosine oligomers obtained by TMG-mediated polymerization of Me-NNTA (Condition: $[M]_0$=1.0 M, $[M]_0$:$[TMG]_0$=10:1, 25 °C in CD_2Cl_2). The spectrum was collected in CD_2Cl_2 solvent.

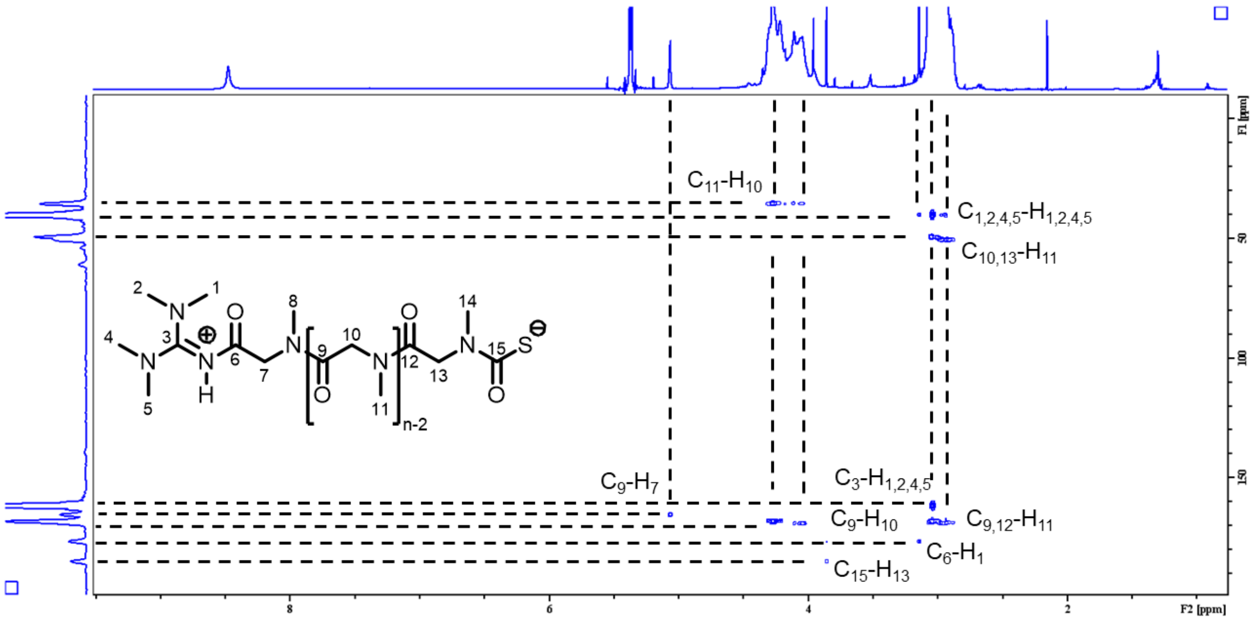

Figure S10. ^{1}H-^{13}C HMBC NMR spectrum of polysarcosine oligomers obtained by TMG-mediated polymerization of Me-NNTA (Condition: $[M]_0$=1.0 M, $[M]_0$:$[TMG]_0$=10:1, 25 °C in CD_2Cl_2). The spectrum was collected in CD_2Cl_2 solvent.

Table S2: Summary of polymerization rate constant (k_p) of ROPs of Me-NNTA or Me-NNCA using different initiators or under different conditions (temperature or solvent)

Entry #	Monomer	Initiator	Solvent	Temperature (°C)	k_p (10^{-3} $M^{-1}s^{-1}$)
1	Me-NNCA	$BnNH_2$	NMP	20	28.8 [S3]
2	Me-NNTA	$BnNH_2$	ACN	70	31.7 [S4]
3	Me-NNTA	$^nBuNH_2$	CH_2Cl_2	25	11.2
4	Me-NNTA	TMG	CH_2Cl_2	25	23.1

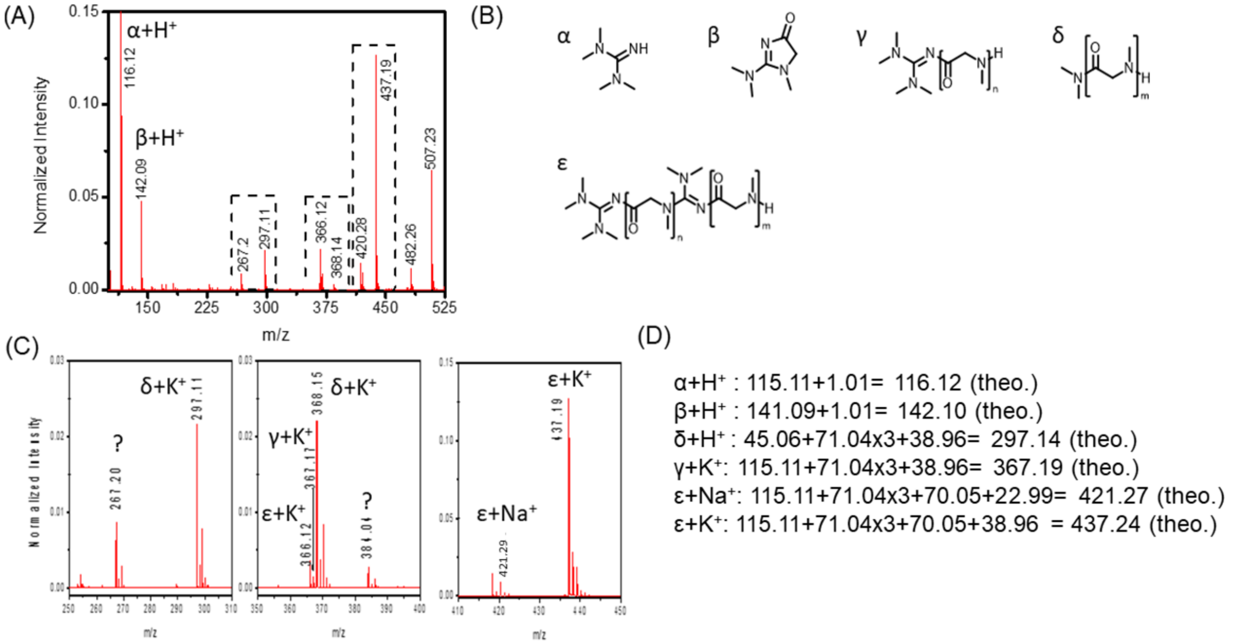

Figure S11. (A) Full and (C) expanded ESI MS spectra of the reaction mixture of Me-NNTA and TMG in 1:1 molar ratio at 25 °C in CD_2Cl_2 together with (B) the chemical structure corresponding to the various mass ions observed in MS spectrum. (D) A comparison of m/z values of selected mass ions in the MS spectrum with the theoretical values based on the chemical structures in (C).

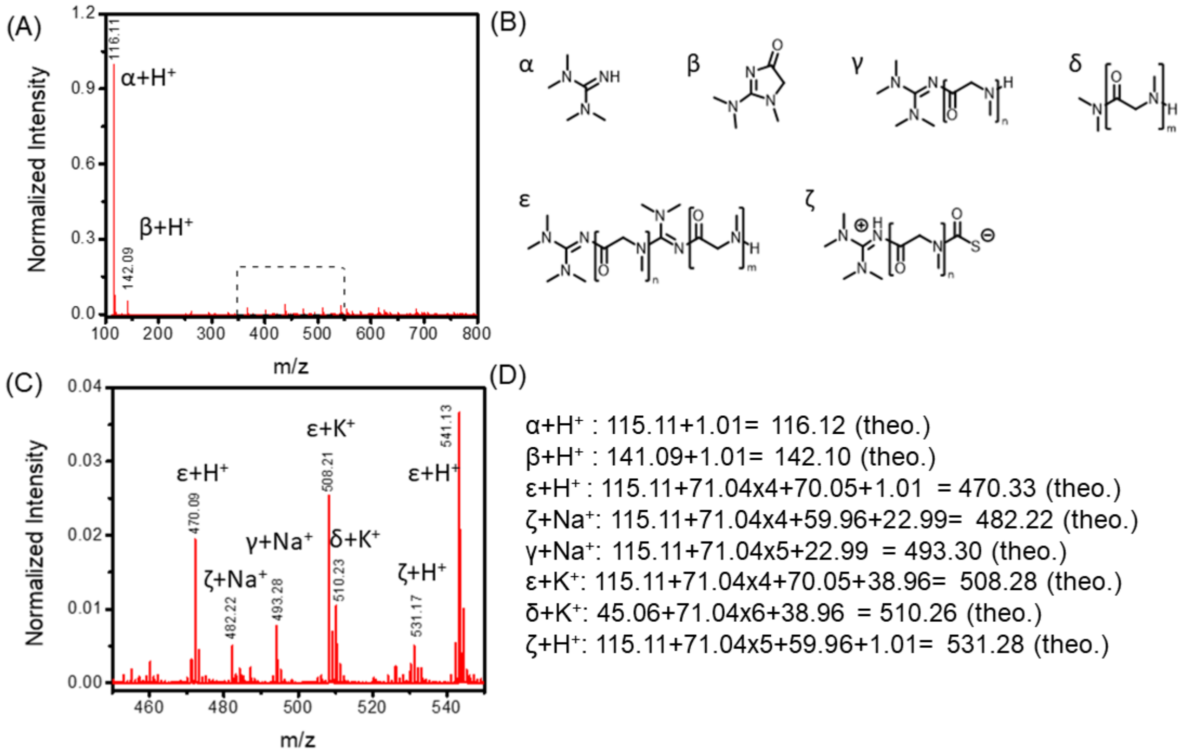

Figure S12. (A) Full and (C) expanded ESI MS spectra of the reaction mixture of Me-NNTA and TMG in 5:1 molar ratio at 25 °C in CD_2Cl_2 together with (B) the chemical structure corresponding to the various mass ions observed in MS spectrum. (D) A comparison of m/z values of selected mass ions in the MS spectrum with the theoretical values based on the chemical structures in (C).

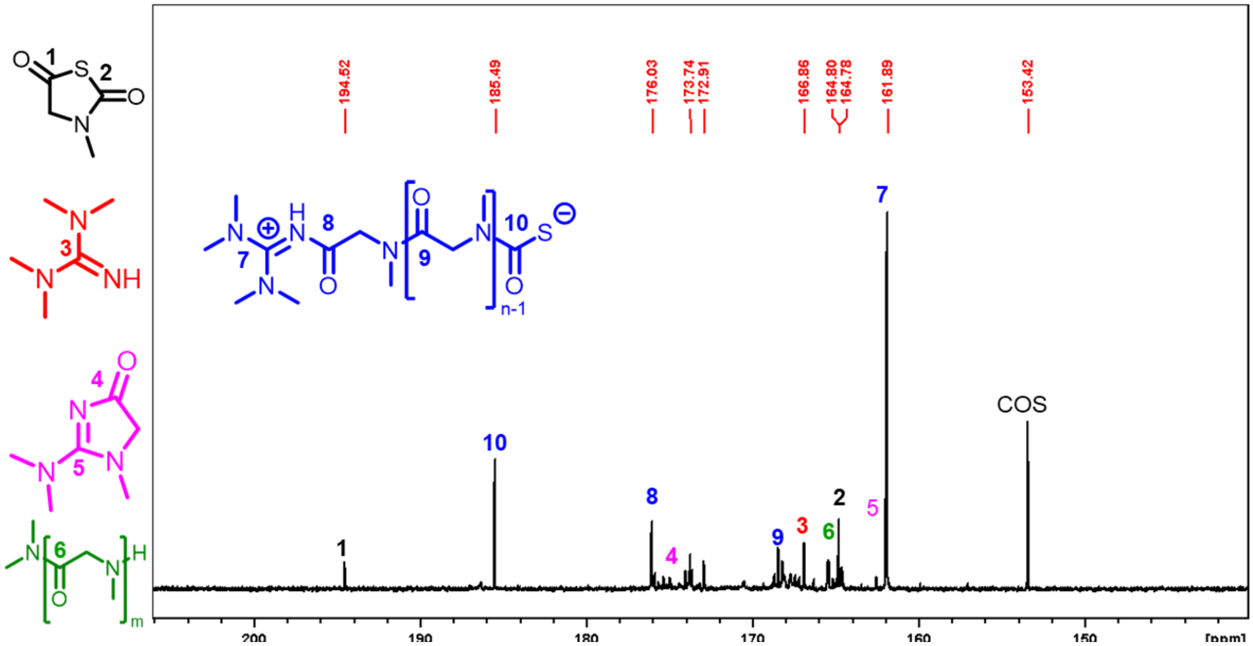

Figure S13. ^{13}C{^{1}H} NMR spectrum of the reaction mixture of Me-NNTA and TMG in 1:1 molar ratio at 25 °C in CD_2Cl_2. ^{13}C signal assignment is based on comparison with known compounds or analogous structures reported in the literature.[S5-S7]

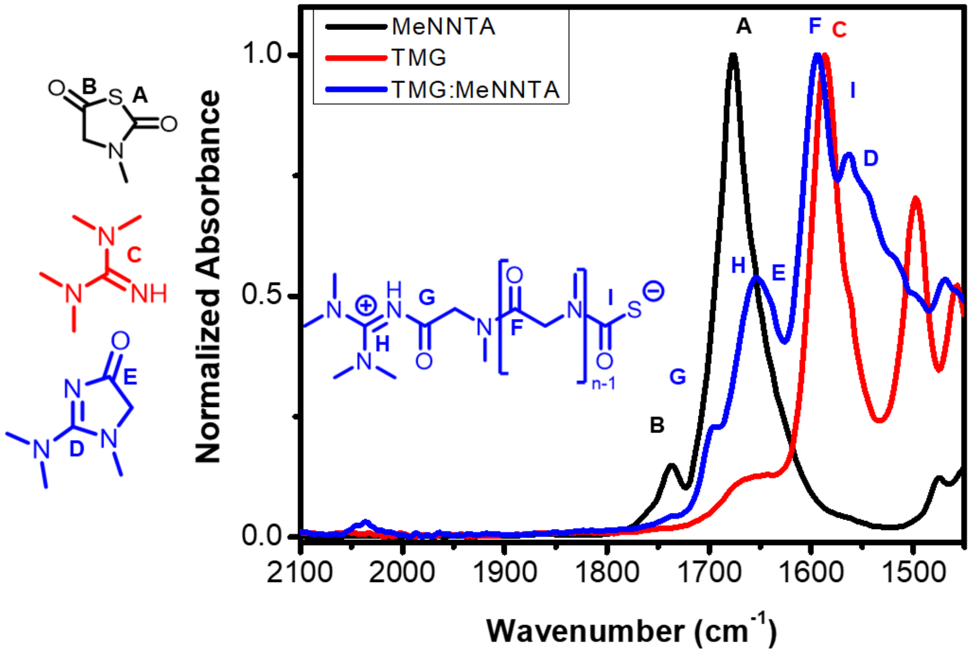

Figure S14. FTIR spectra of Me-NNTA, TMG, and the reaction mixture of Me-NNTA and TMG in 1:1 molar ratio at 25 °C in CD_2Cl_2. FTIR peak assignment compares known compounds or analogous structures reported in the literature.[S6, S8, S9]

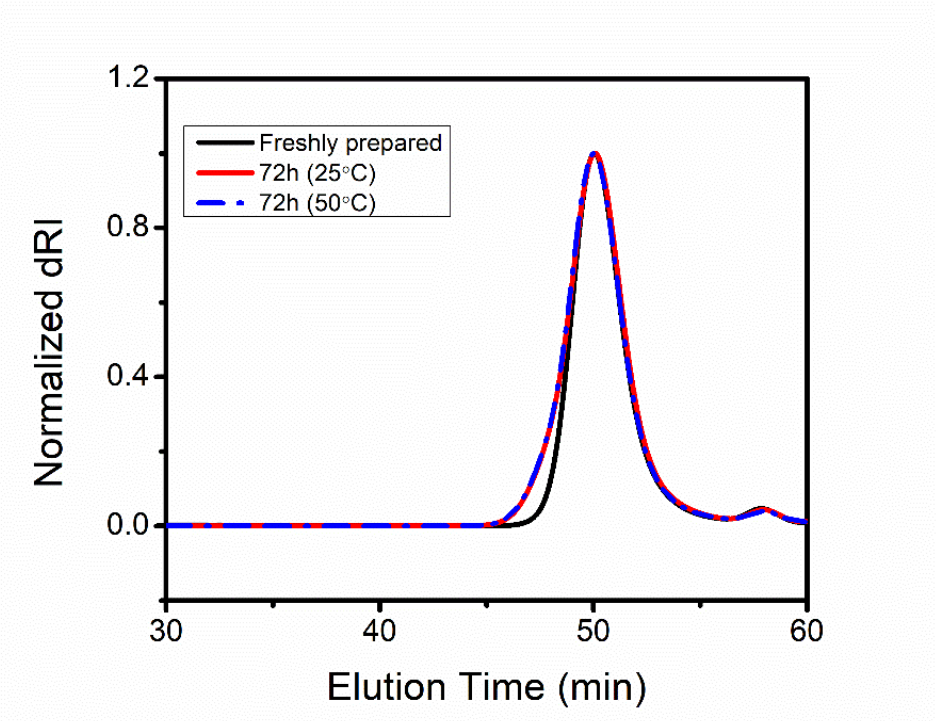

Figure S15. Representative SEC chromatograms of polysarcosine polymers obtained by TMG-mediated ROP of Me-NNTA (Conditions: $[M]_0$= 1.0 M, $[I]_0$= 8.33 mM, $[M]_0$:$[I]_0$=120:1, 25 °C in CH_2Cl_2) obtained 1 h after reaching quantitative conversion in 6 h (−) and after being stirred at 25 °C (−) or 50 °C (---) for additional 72 h.

Table S3. Summary of M_n and PDI results of polysarcosine polymers determined by SEC-MALS-DRI analysis (**Figure S15**). [a]

Entry #	polysarcosine samples	M_n (Theo.) [b] (kg/mol)	M_n (SEC) [c] (kg/mol)	Đ [c]
1	Freshly prepared	8.6	8.5	1.02
2	25°C (72h)	8.6	10.5	1.08
3	50°C (72h)	8.6	10.2	1.07

[a.] The freshly prepared polysarcosine was obtained by taking aliquots of the reaction mixture from the TMG-mediated polymerization of Me-NNTA at 25 °C in CH_2Cl_2 ($[M]_0$=1.0 M, $[I]_0$=8.33 mM, $[M]_0$:$[TMG]_0$ = 120:1) obtained 1 h after reaching quantitative conversion in 6 h. The remaining reaction mixture was stirred at 25 °C or 50 °C for an additional 72 h before SEC analysis. [b.] Theoretical molecular weights were calculated from the $[M]_0$:$[TMG]_0$ ratios and conversion; [c.] Experimental molecular weight and polydispersity index were determined by the SEC-MALS-DRI method using dn/dc = 0.230 mL/g in HFIP/CF_3CO_2K (3 mg/mL) at 40 °C.

Present address:

[#]D.S: Sorbonne university, Paris, France, 75006
[$]B.A.C: Duane Morris LLP, New York, NY, United States 10036
[&]J.-W. N: Brown University, Providence, RI, United States 02912

References and Notes

S1. Cao, J.; Siefker, D.; Chan, B. A.; Yu, T.; Lu, L.; Saputra, M. A.; Fronczek, F. R.; Xie, W.; Zhang, D., Interfacial Ring-Opening Polymerization of Amino-Acid-Derived N-Thiocarboxyanhydrides Toward Well-Defined Polypeptides. *ACS Macro Letters* **2017,** *6* (8), 836-840.
S2. Kricheldorf, H. R.; Sell, M.; Schwarz, G., Primary Amine ‐ Initiated Polymerizations of α–Amino Acid N ‐ Thiocarbonic Acid Anhydrosulfide. *J. Macro. Sci. Part A* **2008,** *45* (6), 425-430.
S3. Fetsch, C.; Grossmann, A.; Holz, L.; Nawroth, J. F.; Luxenhofer, R., Polypeptoids from N-Substituted Glycine N-Carboxyanhydrides: Hydrophilic, Hydrophobic, and Amphiphilic Polymers with Poisson Distribution. *Macromolecules* **2011,** *44* (17), 6746-6758.
S4. Tao, X.; Zheng, B.; Kricheldorf, H. R.; Ling, J., AreN-substituted glycineN-thiocarboxyanhydride monomers really hard to polymerize? *J. Polym. Sci., Part A: Polym. Chem.* **2017,** *55* (3), 404-410.
S5. G. L. Rowley, G. L. K., The conversion of isotopically labeled glycine to 1-methyl-2-amino-2-imidazolin-4-one (creatinine). *J. Heterocycl. Chem.* **1972,** *9* (2), 203-205.
S6. Yu, T.; Yamada, T.; Weiss, R. G., In situ Formation of Thermally Stable, Room-Temperature Ionic Liquids from CS2and Amidine/Amine Mixtures. *Chem. Mater.* **2010,** *22* (19), 5492-5499.
S7. Kenyon, G. L.; Rowley, G. L., Tautomeric preferences among glycocyamidines. *J. Am. Chem. Soc.* **1971,** *93* (21), 5552-5560.
S8. Guo, L.; Lahasky, S. H.; Ghale, K.; Zhang, D., N-Heterocyclic Carbene-Mediated Zwitterionic Polymerization of N-Substituted N-Carboxyanhydrides toward Poly(α-peptoid)s: Kinetic, Mechanism, and Architectural Control. *J. Am. Chem. Soc.* **2012,** *134* (22), 9163-9171.
S9. Rowley, G. L.; Greenleaf, A. L.; Kenyon, G. L., On the specificity of creatine kinase. New glycocyamines and glycocyamine analogs related to creatine. *J. Am. Chem. Soc.* **1971,** *93* (12), 5542-5551.
